# Intravascular ultrasound analysis of spontaneous pulmonary artery lumen enlargement after balloon pulmonary angioplasty in patients with chronic thromboembolic pulmonary hypertension

**DOI:** 10.3389/fmed.2026.1899355

**Published:** 2026-07-30

**Authors:** Wande Yu, Yang Luo, Shifa Zhu, Yuxing Lai, Minghui Zhu, Xingyue Chang, Hang Zhang

**Affiliations:** 1 Division of Cardiology, Nanjing First Hospital, Nanjing Medical University, Nanjing, China; 2 3rd College, Nanjing Medical University, Nanjing, China; 3 Division of Cardiology, Ganyu District Hospital, Lianyungang, Jiangsu, China; 4 Division of Cardiology, The First Affiliated Hospital of Fujian Medical University, Fuzhou, Fujian, China

**Keywords:** balloon pulmonary angioplasty, chronic thromboembolic pulmonary hypertension, intravascular ultrasound, nitrate-nitrite, pulmonary artery lumen

## Abstract

**Background:**

Balloon pulmonary angioplasty (BPA) is an important therapeutic strategy for chronic thromboembolic pulmonary hypertension (CTEPH) requiring staged procedures. Notably, incompletely expanded pulmonary artery lumens enlarge spontaneously between sessions, but the mechanism remains unclear.

**Objectives:**

To investigate the morphological mechanisms of spontaneous lumen enlargement after BPA and their association with vasoactive mediators.

**Methods:**

A total of 151 pulmonary artery segments from 92 BPA procedures in 24 CTEPH patients were included for analysis. Intravascular ultrasound (IVUS) was used to assess the same vascular segments immediately after the first BPA procedure and again before the second BPA procedure. Plasma levels of vasoactive mediators were also measured in the corresponding pulmonary artery segments at both time points.

**Results:**

Before the second procedure, IVUS showed significant increases in lumen area (LA, 10.2 → 16.8 mm^2^, *p* < 0.001) and vascular area (VA, 16.4 → 25.5 mm^2^, *p* < 0.001), and a reduction in lesion burden (LB, 35.9% → 32.1%, *p* < 0.001). Linear mixed-effects model analysis showed that changes in VA and LB were independently associated with the change in LA. Plasma nitrate-nitrite (NOx) levels were higher and plasma endothelin-1 concentrations were lower before the second procedure compared with immediately after the first procedure. Mixed-effects model analysis showed that only plasma NOx difference was independently associated with LA difference.

**Conclusion:**

Spontaneous lumen enlargement after BPA was associated with vascular enlargement and reduced lesion burden, suggesting a morphological basis for this phenomenon. Pulmonary vascular enlargement may be associated with increased plasma NOx levels.

## 1 Introduction

With advances in balloon pulmonary angioplasty (BPA), hemodynamics, exercise capacity, and long-term survival have improved substantially in patients with chronic thromboembolic pulmonary hypertension (CTEPH) (1, 2). BPA has therefore become an important treatment option for patients with CTEPH.

CTEPH patients mostly require multiple BPA procedures to achieve a mean pulmonary arterial pressure (mPAP) < 30 mmHg or a pulmonary vascular resistance (PVR) < 3 Wood units (3). To reduce the occurrence of lung injury, small balloons are preferred during the early stages of the BPA procedure, especially in patients with high mPAP (4, 5). Interestingly, during the staged BPA procedures, underexpanded pulmonary artery segments frequently exhibit subsequent lumen enlargement (6). This phenomenon has important clinical implications, as it may influence balloon sizing in subsequent procedures, contribute to continued hemodynamic improvement, and enhance functional capacity. However, the physiological mechanism behind the spontaneous enlargement of pulmonary artery segments observed during pulmonary angiography was unclear.

Revascularizing coronary chronic total occlusions may also undergo spontaneous enlargement during long-term follow-up, which may be attributed to positive remodeling (7, 8). However, the pathogenesis of coronary heart disease and CTEPH is different, and the stenosis or occlusion of the pulmonary artery in CTEPH is caused by chronic thrombus obstruction in the lumen (5, 9). Accordingly, the morphological changes of spontaneous enlargement of the coronary artery cannot be applied to the long-term spontaneous enlargement of the pulmonary artery (PA) in patients with CTEPH. We speculate that it may be related to vascular dilation or/and reduced lesion burden. In order to elucidate the morphological mechanism of this long-term enlargement phenomenon in pulmonary arteries, intravascular ultrasound (IVUS) was used to evaluate the same pulmonary artery segments immediately after the first BPA session and again before the second session. We also measured local plasma concentrations of vasoactive mediators at both time points to explore possible biological correlates of this phenomenon.

## 2 Methods

### 2.1 Study population and study design

This study included patients with CTEPH treated at a participating center of the National Registry and Cohort Study of Pulmonary Vascular Disease (NCPVD), an observational, multicenter, prospective cohort of patients with pulmonary hypertension. The study was registered at ClinicalTrials.gov (NCT05368467; local approval number: NMU20220505). Consecutive patients with CTEPH who underwent BPA at a single center (Nanjing First Hospital) of the NCPVD between December 2024 and January 2025 were screened. Patients with concomitant acute pulmonary embolism were excluded. All patients provided written informed consent, and the protocol was approved by the Ethics Review Committee of Nanjing First Hospital.

During staged BPA treatment, pulmonary artery segments or subsegments that underwent initial balloon dilation were considered for inclusion. IVUS was used to measure lumen area (LA), vascular area (VA), and lesion burden (LB) at the site where the branch vessel joined the parent vessel immediately after balloon dilation during the first procedure. The same site was reassessed 4 to 8 weeks later, before the second procedure. Blood was collected from the target vessel at both time points. Pulmonary hypertension medications (soluble guanylate cyclase agonist, endothelin receptor antagonists and prostacyclin analogs) were kept unchanged between the two procedures.

World Health Organization (WHO) functional class, 6-min walk distance (6MWD), plasma level of brain natriuretic peptide (BNP), cardiopulmonary hemodynamic indices, cardiac index (CI; by the Fick method), and pulmonary vascular resistance (PVR) were determined before and after the first procedure and immediately before the second procedure.

### 2.2 Patient and pulmonary artery segments selection

**Figure 1:**
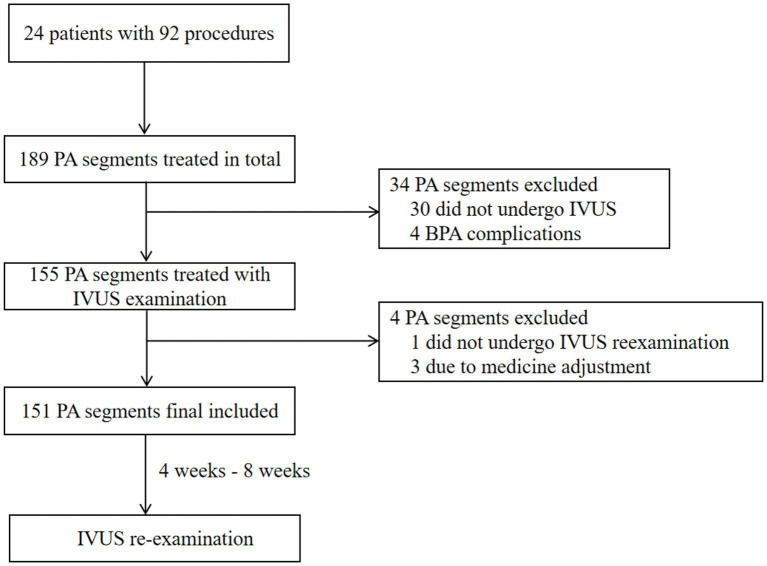
Study flowchart.

A total of 92 BPA procedures were performed in 24 patients with CTEPH (Figure 1). All eligible pulmonary artery segments that underwent balloon dilation during the first BPA procedure were consecutively enrolled for IVUS analysis. Target vessels were subsegmental pulmonary arteries or their further branches that underwent balloon dilation during the first BPA procedure. In each patient, all treatable segments meeting the angiographic inclusion criteria (visible lesion, wire-crossable, no severe tortuosity) were included without selection based on lesion morphology or location. IVUS was attempted in every treated segment; only segments where IVUS could not be safely advanced or where image quality was insufficient for contour tracing were excluded prospectively. Overall, 189 pulmonary artery segments were screened. Thirty segments were excluded because IVUS could not be performed owing to vascular tortuosity or the absence of a visible lesion on angiography. Of the remaining 159 segments, 4 were excluded due to BPA-related complications. During follow-up, 4 segments were excluded because of BPA-related complications, 3 because medications were adjusted between procedures, and 1 because the target segment could not be identified at the second procedure. The final analysis therefore included 151 pulmonary artery segments. All exclusions were based on predefined angiographic criteria (tortuosity or absence of visible lesion) and were applied before IVUS acquisition, thereby avoiding selection bias related to IVUS findings.

### 2.3 BPA procedure

A 6F sheath was inserted through the femoral vein. A 6F Judkins Right 4.0 guiding catheter was advanced into the main pulmonary artery over a 0.025-inch hydrophilic guidewire. After the lesion was crossed with a 0.014-inch wire, balloon size was selected on the basis of selective pulmonary angiography. The balloon was inflated to nominal pressure. After dilation, use a guiding catheter to record the pressure proximal to the target vessel. IVUS measurements were performed at the junction where the target vessel merged with the main branch vessel, and the same site was measured at both time points (immediately after the first BPA procedure and before the second BPA procedure). Right heart catheterization was performed before the first procedure and again immediately before the second procedure.

### 2.4 IVUS image

**Figure 2:**
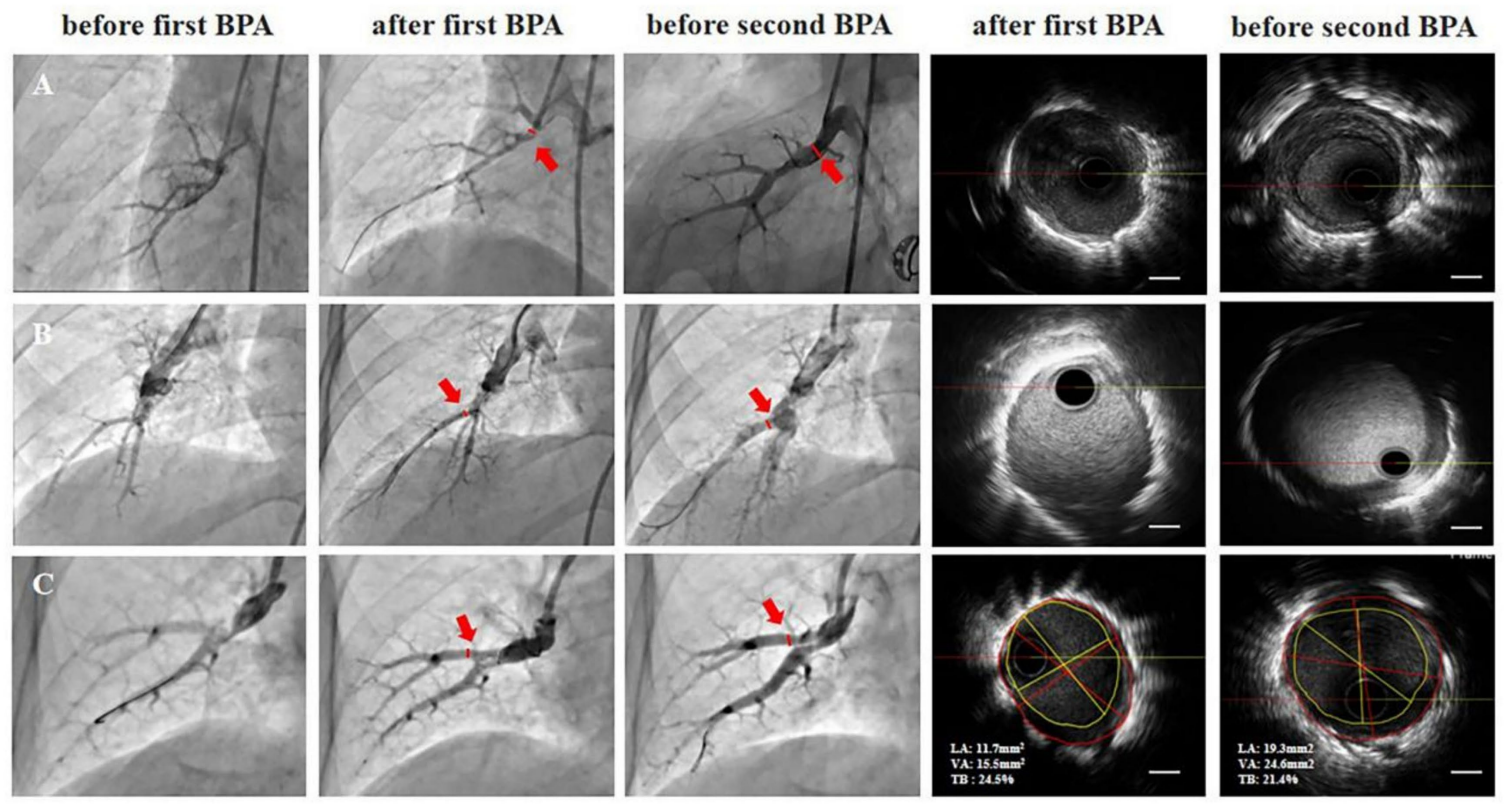
Representative angiographic and IVUS images between first and second BPA procedure. **(A–C)** Represent three pulmonary artery segments of three independent patients, respectively. The first and second columns show the pulmonary angiography images before and immediately after the first BPA procedure. The third column shows the pulmonary angiography images before the second BPA procedure. The fourth and fifth columns show IVUS images (corresponding to the red arrow in the second and third columns) immediately after the first BPA procedure and before the second BPA procedure. In case **C**, the external elastic membrane (vascular area, VA) is indicated by the red line, and the lumen area (LA, the abrupt transition from the echo-lucent zone to the area with thrombus) is indicated by the yellow line. Lesion burden (LB) was calculated as (VA - LA)/VA.

IVUS imaging was performed using a 60-MHz AVVIGO+ Multi-Modality Guidance System (Boston Scientific, China). The IVUS catheter was advanced to the distal portion of the target branch vessel and then pulled back to the main branch vessel at 5 mm/s. Because many lesions showed a honeycomb-like structure, LA was defined by tracing the lumen contour at the guidewire location after balloon dilation. VA was defined as the area circumscribed by the adventitial border of the pulmonary artery. LB was calculated as (VA - LA)/VA and was used as an index of lesion burden (Figure 2). Vascular diameter was measured at the same site as VA and LA, defined as the maximum vascular diameter measured from the IVUS cross-sectional image at the target segment. Measurements were performed independently by two experienced observers.

### 2.5 Plasma samples and enzyme-linked immunosorbent assay

Because lesion type, thrombus burden, and perfusion status may vary across pulmonary artery segments, blood samples were collected from each target segment immediately after balloon dilation during the first procedure and again before the second balloon dilation. Samples were collected in ethylenediaminetetraacetic acid tubes and centrifuged at 1500 g for 15 min. Plasma was separated and stored at −80\u00B0C until analysis.

Plasma norepinephrine (NE), endothelin-1 (ET-1), and prostaglandin E2 (PGE2) concentrations were measured by enzyme-linked immunosorbent assay according to the manufacturers’ instructions (Elabscience, Wuhan, China). Nitrate-nitrite (NOx) concentration was measured with a total nitric oxide assay kit (Beyotime Biotechnology, Guangzhou, China).

### 2.6 Statistical analysis

Continuous variables are presented as mean ± standard deviation (SD) or median with interquartile range (IQR), and categorical variables as counts (percentages). Paired comparisons between the two time points (immediately after the first BPA procedure vs. before the second BPA procedure) were performed using paired t-tests or Wilcoxon signed-rank tests, as appropriate.

To account for repeated measurements of the same pulmonary artery segments, linear mixed-effects models were used as the primary analysis. Patient and segment identifiers were included as random intercepts. Time point (post-first BPA vs. pre-second BPA) was entered as a fixed effect, along with VA, LB, and other covariates. Interaction terms (Time × VA, Time × LB) were tested to evaluate whether the effects of VA and LB on LA differed between the two time points. A separate model examined predictors of percentage change in LA. For each pulmonary artery segment, the change (*Δ*) of LA and plasma mediators (NOx, ET-1, NE, PGE2) between the two time points was calculated as follows:.

Δ = (Value_pre-second BPA - Value_post-first BPA)

Model assumptions (linearity, normality of residuals, homoscedasticity) were verified using residual plots. Regression coefficients (B) with 95% confidence intervals (CI) are reported. All statistical tests were two-sided, and a *p* value < 0.05 was considered statistically significant. Sensitivity analyses using false discovery rate (FDR) correction were performed for the primary IVUS outcomes. Analyses were performed using SPSS version 24.0 (IBM Corp., Armonk, NY, USA) or R software (version 4.2.0, lme4 package).

## 3 Results

### 3.1 Baseline characteristics

Overall, 151 (79.9%) pulmonary artery segments from 92 BPA procedures in 24 patients were consecutively included in the final analysis after applying predefined angiographic and IVUS image quality criteria. Baseline demographic and disease characteristics of the first BPA procedure are shown in Table 1. Among the 151 analyzed segments, the median number of segments per patient was 6 (interquartile range: 4–9), and the median number of segments per procedure was 2 (interquartile range: 1–3). The mean age was 69.9 ± 5.9 years, and 25% were male. The time interval between the first BPA procedure and the second BPA procedure was a mean of 48.6 ± 18.2 days. The ratio of tricuspid annular systolic plane excursion (TAPSE) to pulmonary artery systolic pressure (PASP) before the first 92 BPA procedures was 0.20 ± 0.14 mm/mmHg. During the interval between two BPA procedures, patients mainly received monotherapy (26.1%) or dual therapy (63.0%). No dose adjustments or medication switches occurred between the first and second BPA procedures.

**Table 1:** Clinical and hemodynamic parameters of the first procedures.

Baseline characteristics	Patient’s parameters
Male sex, n (%)	6 (25%)
Age, y	69.9 ± 5.9

	Characteristics of first BPA procedure
Interval time (Days)	48.6 ± 18.2
TAPSE/PASP (mm/mmHg)	0.20 ± 0.14
Medical therapy	
Mono (*n*, %)	24 (26.1)
Dual (*n*, %)	58 (63.0)
Triple (*n*, %)	10 (10.9)

	Before first procedure	After first procedure	*P*
WHO FC	III	II	0.005
I-II (*n*, %)	28 (30.4)	54 (58.7)	
III (*n*, %)	61 (66.3)	38 (41.3)	
IV (*n*, %)	3 (3.3)	0 (0)	
6MWD (m)	278 ± 107	322 ± 99	<0.001
BNP (pg/mL)	653.4 ± 997.7	430.9 ± 674.1	0.043
RAP (mmHg)	12.9 ± 5.5	11.9 ± 5.8	0.023
sPAP (mmHg)	79.1 ± 17.6	70.0 ± 15.8	<0.001
mPAP (mmHg)	46.9 ± 8.6	41.7 ± 8.1	<0.001
dPAP (mmHg)	29.8 ± 5.1	27.3 ± 5.7	0.006
CI (L/min/m2)	2.3 ± 0.5	2.5 ± 0.5	0.031
TPR (Wood unit)	12.3 ± 3.8	10.2 ± 2.9	<0.001

TAPSE: tricuspid annular plane systolic excursion, WHO FC: World Health Organization Functional class, PASP: pulmonary artery systolic pressure, 6MWD: 6-min walking distance, BNP: brain natriuretic peptide, sPAP: systolic pulmonary arterial pressure, mPAP: mean pulmonary arterial pressure, dPAP: diastolic pulmonary arterial pressure, CI: cardiac index, TPR: total pulmonary resistance.

There were significant improvements in WHO functional class, 6-min walk distance, and BNP levels compared to those before the BPA procedure. All the hemodynamic parameters were also improved after the BPA procedure (Table 1). Moreover, mPAP and TPR were decreased, and cardiac index (CI) was increased immediately after the first BPA procedure compared to before the second BPA procedure (Table 2).

**Table 2:** Hemodynamic parameters immediately after first procedure and before second procedure.

Hemodynamic parameters	After first procedure	Before second procedure	*P*
RAP (mmHg)	11.9 ± 5.8	11.4 ± 4.9	0.757
sPAP (mmHg)	70.0 ± 15.8	68.3 ± 18.1	0.160
mPAP (mmHg)	41.7 ± 8.1	40.7 ± 8.8	0.012
dPAP (mmHg)	27.3 ± 5.7	26.3 ± 5.7	0.216
CI (L/min/m2)	2.5 ± 0.5	2.7 ± 0.7	0.026
TPR (Wood unit)	10.2 ± 2.9	9.4 ± 3.7	0.047

RAP: right atrial pressure, sPAP: systolic pulmonary arterial pressure, mPAP: mean pulmonary arterial pressure, dPAP: diastolic pulmonary arterial pressure, CI: cardiac index, TPR: total pulmonary resistance.

### 3.2 Lesion and procedural characteristics

The 151 analyzed pulmonary artery segments included ring-like stenoses, web lesions, subtotal occlusions, and total occlusions, with occlusive lesions accounting for the largest proportion (57.6%; Table 3). The median balloon diameter during the first BPA procedure was 3.0 mm (interquartile range, 3.0–4.0 mm). The mean vascular diameter measured by IVUS after the first procedure was 4.5 ± 0.9 mm. Proximal mean pulmonary arterial pressure was also recorded to explore whether local pressure influenced subsequent spontaneous lumen enlargement (Table 3).

**Table 3:** Lesion and procedural characteristics.

Lesion type	Number (%)
Ring-like stenosis lesion	15 (9.9)
Web lesion	49 (32.5)
Subtotal occlusion lesion	52 (34.4)
Total occlusion lesion	35 (23.2)
Median balloon size (mm)	3.0 (3.0–4.0)
Vascular diameter (mm)	4.5 ± 0.9
Proximal mean PAP (mmHg)	37.4 ± 8.5

### 3.3 Changes of lumen area, vascular area, and lesion burden based on IVUS

**Figure 3:**
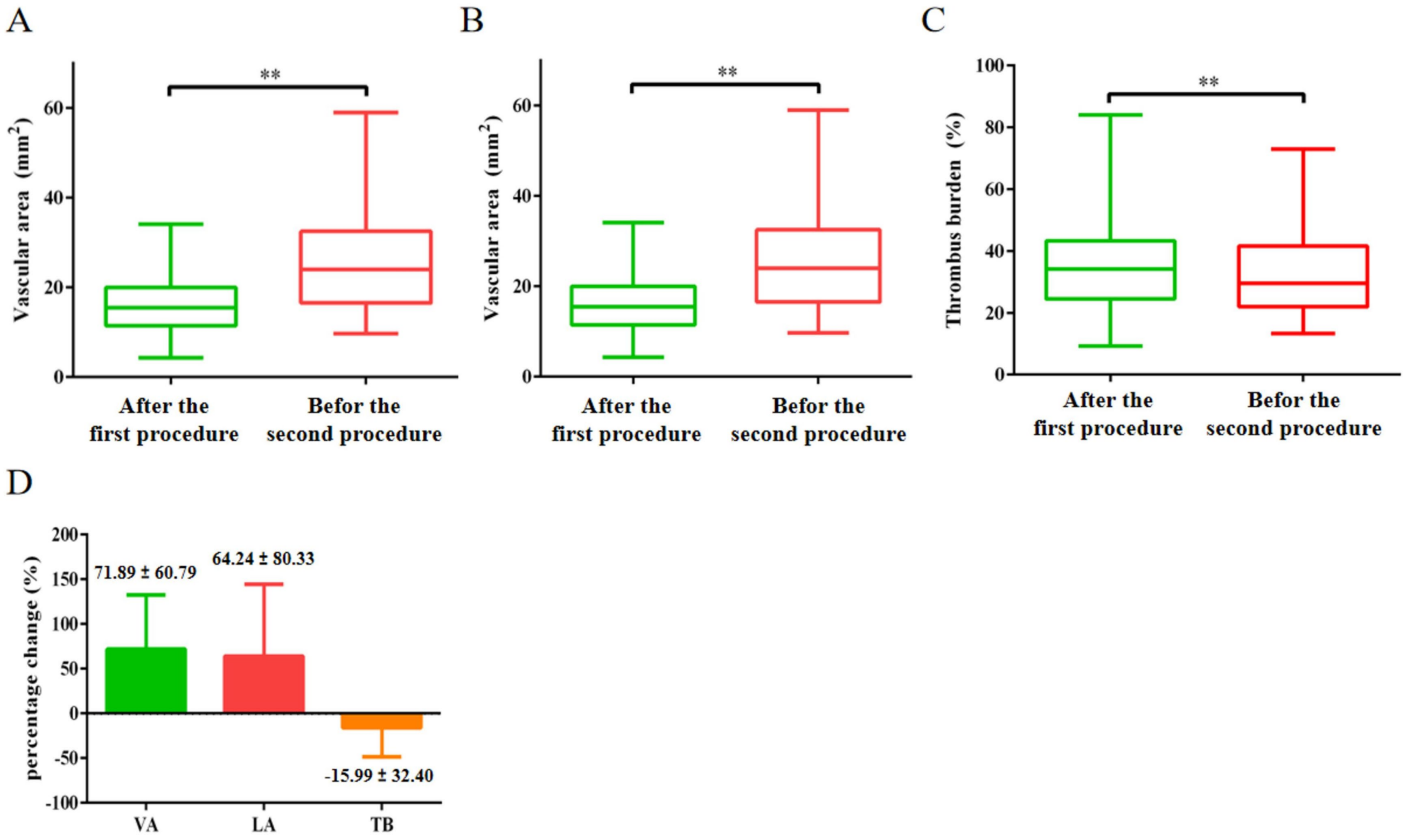
Changes in lumen area, vascular area, and lesion burden based on IVUS. **(A)** Changes in lumen area quantified by IVUS. **(B)** Changes in vascular area quantified by IVUS. **(C)** Changes in lesion burden quantified by IVUS. **(D)** The percentage change in lumen area, vascular area and lesion burden. ** *p* < 0.01.

Before the second procedure, LA, VA, and LB were measured again at the same pulmonary artery site. Compared with measurements obtained immediately after balloon dilation during the first procedure, both LA and VA increased significantly before the second procedure. LA increased from 10.2 ± 4.5 to 16.8 ± 7.9 mm2, corresponding to a 1.7-fold increase (95% CI, 1.5–1.9; *p* < 0.001; Figure 3A). VA increased from 16.4 ± 6.9 to 25.5 ± 11.6 mm2, corresponding to a 1.6-fold increase (95% CI, 1.4–1.9; *p* < 0.001; Figure 3B). In contrast, LB decreased from 35.9 ± 14.3% to 32.1 ± 12.9% (95% CI, 0.9–1.0 fold change; *p* < 0.001; Figure 3C). After FDR correction, all three comparisons remained statistically significant (q < 0.001). The magnitude of LA increase was greater than the magnitude of LB reduction when percentage changes were compared (Figure 3D), suggesting that vascular expansion contributes more to lumen enlargement than lesion regression alone.

To identify factors independently associated with the change in LA, a linear mixed-effects model was constructed with patient and segment as random intercepts. As shown in Table 4, both VA (B = 0.63, 95% CI: 0.60–0.67, *p* < 0.001) and LB (B = −0.22, 95% CI: −0.25 to −0.19, p < 0.001) were significantly associated with LA. Variance inflation factor (VIF) was 3.42 for VA and 3.58 for LB, indicating that collinearity between the two predictors was within an acceptable range (VIF < 5). Therefore, both variables contributed independently to the model. Significant interactions were observed for both Time × VA (B = −0.08, *p* < 0.001) and Time × LB (B = 0.04, *p* = 0.001), indicating that the effects of VA and LB on LA differed between the two time points. Specifically, the positive effect of VA on LA increased from 0.55 at post-first BPA to 0.63 at pre-second BPA, while the negative effect of LB on LA intensified from −0.18 to −0.22. Balloon size and proximal mean pulmonary artery pressure were not significantly associated with LA (both *p* > 0.05).

**Table 4:** Linear mixed-effects model for lumen area.

Fixed effects	B	95% CI	t	*P*
Intercept	6.89	3.99 to 9.78	4.78	<0.001
Main effects				
VA, mm^2^	0.63	0.60 to 0.67	35.66	<0.001
LB, %	−0.22	−0.25 to −0.19	−15.05	<0.001
Balloon size, mm	0.29	−0.16 to 0.75	1.28	0.205
Proximal mean PAP, mmHg	−0.002	−0.05 to 0.05	−0.07	0.949
Interaction with time (post-first BPA vs. pre-second BPA)				
Time × VA	−0.08	−0.13 to −0.04	−3.69	<0.001
Time × LB	0.04	0.02 to 0.06	3.46	0.001

CI: confidence interval; PAP: pulmonary artery pressure; BPA: balloon pulmonary angioplasty; VA: vascular area; LB: lesion burden. Variance inflation factor (VIF) was 3.42 for VA and 3.58 for LB, indicating acceptable collinearity. Random intercepts were included for patient and segment.

### 3.4 Changes of plasma NOx, NE, ET-1, and PGE2

**Figure 4:**
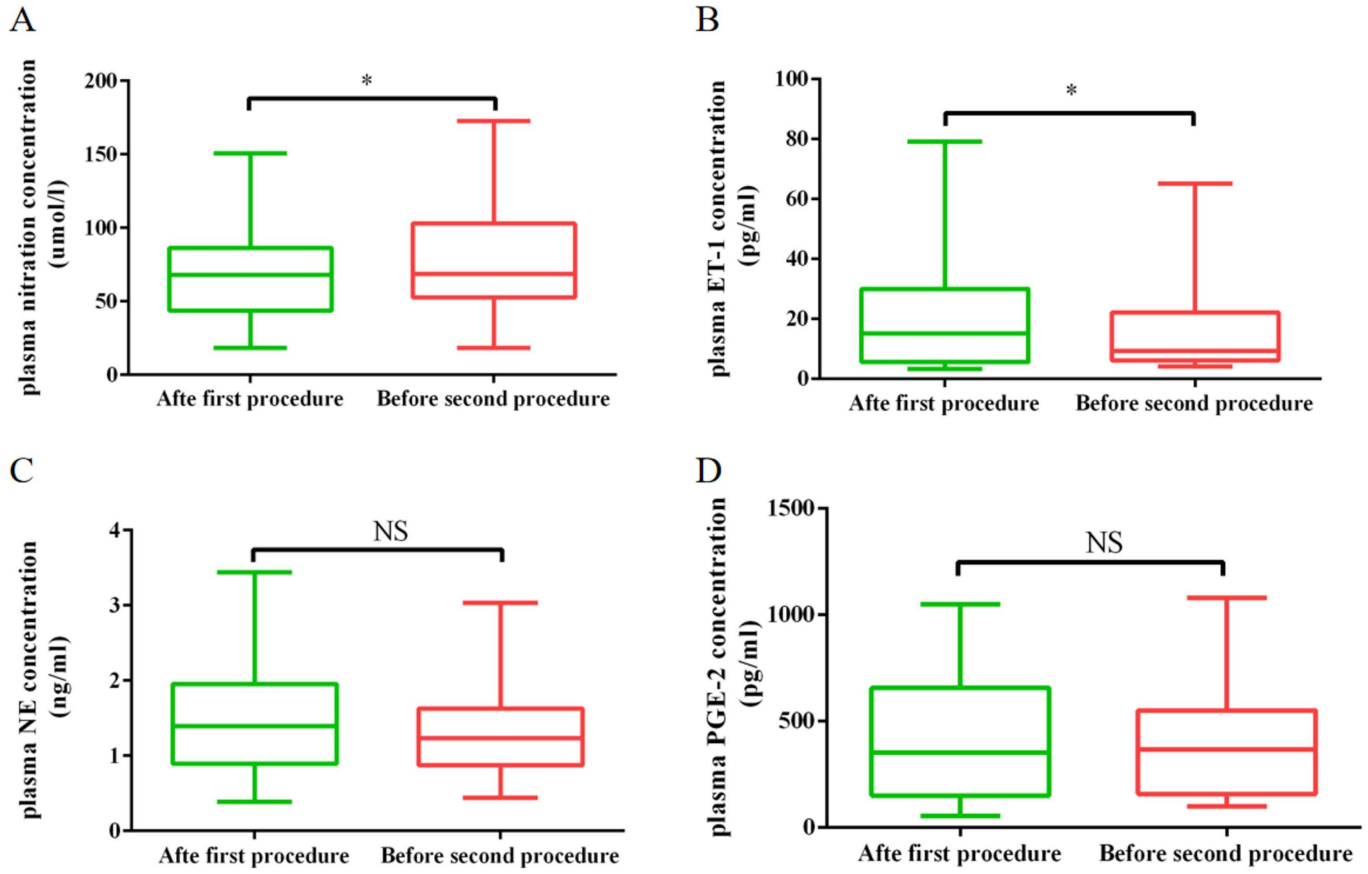
Changes in plasma NOx, NE, ET-1, and PGE2 between immediately after first procedure and before the second procedure. **(A)** Changes of plasma NOx levels. **(B)** Changes of plasma ET-1 concentration. **(C)** Changes of plasma NE concentration. **(D)** Changes of plasma PGE-2 concentration. *p < 0.05. NS, not significant.

Because vasoactive mediators contribute to pulmonary vasomotor tone and vascular remodeling (10), we measured their concentrations in plasma sampled from the target pulmonary artery. Before the second procedure, plasma NOx levels were higher than those measured immediately after the first procedure (70.93 ± 34.82 vs. 79.91 ± 34.86 umol/L, *p* = 0.037), whereas plasma ET-1 concentrations were lower (20.91 ± 19.05 vs. 16.72 ± 16.67 pg./mL, *p* = 0.043). No significant differences were observed in NE or PGE2 concentrations between the two time points (Figure 4). However, mixed-effects model showed that only change in NOx levels and change in LA (B = 0.14, 95% CI: 0.02–0.26, *p* = 0.019) (Table 5).

**Table 5:** Mixed-effects model for factors associated with change in lumen area.

Fixed effects	B	95% CI for B	*t*	*P*
Intercept	51.48	2.75 to 100.21	2.09	0.039
Vasoactive mediators				
Δ Plasma NOx (umol/l)	0.24	0.12 to 0.35	2.36	0.019
Δ Plasma ET-1 (pg/ml)	−0.09	−0.19 to −0.01	−1.81	0.073
Δ Plasma NE (ng/ml)	−0.08	−0.29 to 0.12	−0.81	0.419
Δ Plasma PGE2 (pg/ml)	−0.04	−0.14 to 0.07	−0.72	0.473
Covariates				
Balloon size (mm)	0.59	−0.29 to 1.47	1.32	0.189
Proximal mean PAP (mmHg)	−2.7	−10.01 to 4.59	−0.73	0.464

CI, confidence interval; NOx, nitrate-nitrite; ET-1, endothelin-1; NE, norepinephrine; PGE2, prostaglandin E2; PAP, pulmonary artery pressure.

## 4 Discussion

In this study, IVUS objectively documented and quantified spontaneous pulmonary artery lumen enlargement after BPA. The increase in LA was accompanied by expansion of VA and reduction in LB, along with a decrease in mPAP and an increase in CI. Using serial IVUS measurements and linear mixed-effects models, we observed that both VA and LB are independently associated with LA enlargement. We also observed higher NOx levels and lower ET-1 concentrations before the second procedure than immediately after the first procedure. However, only percentage of plasma NOx difference was independently associated with percentage of LA difference.

The present study provides direct morphological evidence for the mechanisms underlying spontaneous pulmonary artery lumen enlargement after BPA. Using serial IVUS measurements and linear mixed-effects models, we found that both VA and LB are independently associated with LA enlargement. More importantly, significant time interactions were observed for both VA and LB, indicating that their effects on LA are not static but intensify over time. Specifically, the positive effect of VA on LA increased from 0.55 at post-first BPA to 0.63 at pre-second BPA, while the negative effect of LB on LA intensified from −0.18 to −0.22. This temporal changes suggest that the contribution of vascular remodeling to lumen enlargement may increase over time.

The pulmonary artery is part of a low-pressure circulation and differs structurally and hemodynamically from the coronary artery (11). In addition, the pathophysiologic mechanisms of CTEPH differ fundamentally from those of coronary chronic total occlusion (12, 13). Therefore, late spontaneous enlargement of pulmonary artery segments after BPA should not be interpreted simply as the pulmonary counterpart of positive remodeling after coronary recanalization (14, 15). Our findings instead support a lesion-specific process involving both vascular expansion and reduction in lesion burden.

Regardless of lesion type, spontaneous enlargement was observed across the treated pulmonary artery segments, and the degree of enlargement did not appear to depend strongly on balloon diameter. Previous work has shown that the pressure gradient across the lesion may continue to improve after BPA (16). Besides, some studies have shown that BPA can lead to sustained improvement in pulmonary hemodynamics and right ventricular function (17, 18). Our findings are consistent with this pattern, because mPAP and TPR continued to decrease and CI continued to increase between procedures. These phenomena may be associated with spontaneous enlargement of pulmonary artery segments, although proximal pressure was not independently associated with VA change in our regression model.

Reoxygenation after BPA, especially in previously occluded lesions, may also alter the local balance of vasoactive mediators within the pulmonary circulation. Nitric oxide, as a vasodilator, mediated signaling pathway plays an important protective role in lung ischemia–reperfusion injury. Recoupling endothelial nitric oxide synthase ameliorates pulmonary vascular remodeling and improves pulmonary arterial hypertension induced by chronic hypoxia (19). The association between change in plasma NOx and change in lumen area is intriguing and suggests that nitric oxide may play a role in spontaneous pulmonary artery enlargement, although further investigation is needed to clarify the nature of this relationship.

This study has several limitations. First, the sample size was small and the study was single-center, limiting generalizability. Second, blood samples were collected under different physiological conditions: immediately after balloon dilation at the first procedure versus before dilation at the second procedure. The acute effects of balloon dilation—including transient changes in shear stress, endothelial activation, and local mediator release—may have influenced NOx and ET-1 levels in the first sample. Third, although mixed-effects models accounted for clustering, residual within-patient correlations cannot be excluded. Fourth, the changes in cardiopulmonary function brought about by spontaneous enlargement of the pulmonary artery after BPA require further exploration. Larger multicenter studies with control groups, longer follow-up, and formal reproducibility analyses are needed to confirm our findings.

## 5 Conclusion

The morphological mechanism of spontaneous pulmonary artery lumen enlargement after BPA involves pulmonary vascular enlargement and reduced lesion burden. Besides, relative increases in plasma NOx levels were associated with lumen enlargement, although causality cannot be established. Larger multicenter studies are needed to confirm these findings and determine their clinical significance.

## Data Availability

The original contributions presented in the study are included in the article/supplementary material, further inquiries can be directed to the corresponding author.
